# Expression of the Adenosine A_2A_-A_3_ Receptor Heteromer in Different Brain Regions and Marked Upregulation in the Microglia of the Transgenic APP_Sw,Ind_ Alzheimer’s Disease Model

**DOI:** 10.3390/biomedicines10020214

**Published:** 2022-01-19

**Authors:** Alejandro Lillo, Iu Raïch, Jaume Lillo, Catalina Pérez-Olives, Gemma Navarro, Rafael Franco

**Affiliations:** 1 Centro de Investigación Biomédica en Red Enfermedades Neurodegenerativas (CiberNed), National Institute of Health Carlos III, Valderrebollo 5, 28031 Madrid, Spain; alillo@ub.edu (A.L.); iraichpa7@ub.edu (I.R.); jaumelillo@ub.edu (J.L.); catalinaperez@ub.edu (C.P.-O.); 2 Department of Biochemistry and Physiology, Faculty of Pharmacy and Food Science, University of Barcelona, 08028 Barcelona, Spain; 3 Department of Biochemistry and Molecular Biomedicine, University of Barcelona, 08028 Barcelona, Spain; 4 Institut de Neurociències, Universitat de Barcelona (UBNeuro), 08036 Barcelona, Spain; 5 School of Chemistry, University of Barcelona, 08028 Barcelona, Spain; 6 Faculty of Biology, Universitat de Barcelona, Diagonal 643, Prevosti Building, 08028 Barcelona, Spain

**Keywords:** neuroinflammation, neurodegenerative disease, dementia, G protein-coupled receptor, activated microglia, purinergic signaling, striatum, hippocampus, cerebral cortex

## Abstract

Adenosine (Ado) receptors have been instrumental in the detection of heteromers and other higher-order receptor structures, mainly via interactions with other cell surface G-protein-coupled receptors. Apart from the first report of the A_1_ Ado receptor interacting with the A_2A_ Ado receptor, there has been more recent data on the possibility that every Ado receptor type, A_1_, A_2A_, A_2B_, and A_3_, may interact with each other. The aim of this paper was to look for the expression and function of the A_2A_/A_3_ receptor heteromer (A_2A_A_3_Het) in neurons and microglia. In situ proximity ligation assays (PLA), performed in primary cells, showed that A_2A_A_3_Het expression was markedly higher in striatal than in cortical and hippocampal neurons, whereas it was similar in resting and activated microglia. Signaling assays demonstrated that the effect of the A_2A_R agonist, PSB 777, was reduced in the presence of the A_3_R agonist, 2-Cl-IB-MECA, whereas the effect of the A_3_R agonist was potentiated by the A_2A_R antagonist, SCH 58261. Interestingly, the expression of the heteromer was markedly enhanced in microglia from the APP_Sw,Ind_ model of Alzheimer’s disease. The functionality of the heteromer in primary microglia from APP_Sw,Ind_ mice was more similar to that found in resting microglia from control mice.

## 1. Introduction

Adenosine (Ado) is a nucleoside that participates in the metabolism of nucleic acids, of enzyme cofactors/substrates (nicotinamide adenine nucleotides, flavin-adenine dinucleotides etc.), and of energy-related relevant molecules, such as ATP. One of the functions of the nucleoside is to be a warning for excess energy expenditure or for hypoxia; in such conditions, ATP is converted to Ado, whose presence activates Ado receptors on the cell surface. There are four Ado receptors whose affinity for Ado varies and that belong to the rhodopsin-like class A G protein-coupled receptors (GPCRs): A_1_, A_2A_, A_2B_, and A_3_. The canonical heteromeric G proteins to which they couple are G_s_ (A_2A_ and A_2B_) and G_i_ (A_1_ and A_3_). Accordingly, activation of the A_2A_R or the A_2B_R leads to increases of [cAMP] and engagement of the protein kinase A-mediated signaling pathway whereas activation of A_1_R or A_3_R leads to decreases in intracellular cAMP levels [1].

GPCRs may form heterodimers and higher-order structures that arise as distinct functional units. Research on Ado receptors has been instrumental to identify a variety of heteromers starting with those established in the striatum with dopamine receptors [2,3]. It was also found that the A_1_ and A_2A_ receptors are sensors of the Ado concentration via the formation of tetrameric structures complexed with two heterotrimeric G proteins, one G_i_ and one G_s_. At low Ado concentrations, G_i_-mediated signaling is predominant whereas at higher concentrations, G_s_-mediated signaling predominates [4,5,6]. More recently, heteromers formed by A_2A_ and A_2B_ and by A_2A_ and A_3_ receptors have been reported, although their physiological function has not yet been fully elucidated [7,8,9].

The A_2A_ receptor (A_2A_R) is enriched in striatal neurons and was largely considered a target for combating Parkinson’s disease. Noteworthy, a first-in-class selective A_2A_R antagonist, istradefylline, has been approved as an adjuvant therapy in Parkinson’s disease [10,11,12,13,14,15]. The receptor is a target for neuroprotection in other neurodegenerative diseases, such as Huntington’s and Alzheimer’s, and in brain injury [16,17,18,19,20,21,22,23,24,25]. In addition, altered expression of (individual) adenosine receptors was found in the brain of AD patients [26]. Importantly, the expression of the receptor in microglia from control samples was fairly low whereas the A_2A_R was markedly upregulated in glial cells in the hippocampus and cerebral cortex of AD patients [26]. A first aim of this paper was to look for the differential expression and functionality of complexes formed by the A_2A_R and the A_3_R (A_2A_A_3_Hets) in different regions of the mice brain.

Age is the main risk factor in AD and, unfortunately, the efforts made to obtain anti-AD drugs have so far failed. Intriguing is the lack of success of therapies based on the benefits provided by a variety of drugs tested in transgenic AD models ([27,28,29] and references therein). A further relevant issue is the lack of appropriate protocols to assess the neuroprotective potential of potential therapeutic drugs in humans [30]. It should, also, be noted that it is doubtful that neurons that progressively die in AD patients can prolong survival by targeting them directly. Alternatively, targeting the glia may be a better option to allow for neuroprotection, especially in those neurological diseases, including AD, in which the microglia are activated [31,32,33].

Acutely, activated proinflammatory and cytotoxic microglial cells are also known as M1. Either by alternative activation pathways or upon phenotypic changes can microglia contribute to repair and regenerate by expressing anti-inflammatory and immune-regulatory molecules (M2a cells) or acquire the M2b/c deactivating phenotype, also expressing anti-inflammatory markers [34]. Activation of the A_2A_R in activated microglia may yet lead to another phenotype, M2d [35,36,37,38]. In this sense, in 2005, we reported iNOS production by primary microglia upon A_2A_R activation [39].

On the one hand, GPCR-mediated signaling is relevant in the activation of microglia occurring in animal models of Alzheimer’s disease (AD) [40]; in fact, GPCRs expressed in microglia could be important for regulating the M1 (proinflammatory)/M2 (neuroprotective) balance in these cells [31,35]. On the other hand, adenosine regulates microglial activation via, at least, A_2A_ and A_3_ receptors [32,41,42,43,44,45,46,47,48,49,50,51,52,53,54,55,56,57]. A second aim of this paper was to look for the differential expression and functionality of the A_2A_A_3_Het in primary microglia from control mice and from the APP_Sw,ind_ AD mouse model, which carries the transgene for the human amyloid precursor protein (APP) with Swedish and Indiana mutations.

## 2. Materials and Methods

### 2.1. Reagents

PSB 777 ammonium salt, 2-Cl-IB-MECA, SCH 58261, and PSB 10 hydrochloride were purchased from Tocris Bioscience (Bristol, UK). Concentrated (10 mM) stock solutions prepared in DMSO were stored at −20 °C. In each experimental session, aliquots of concentrated solutions of compounds were thawed and conveniently diluted in the appropriate experimental solution. Lipopolysaccharides from Escherichia coli O111:B4 (LPS) and Interferon-γ (IFN-γ) were purchased from SigmaAldrich (St. Louis, MO, USA).

### 2.2. APP Transgenic Mouse Model of Alzheimer’s Disease (AD)

APP_Sw,Ind_ transgenic mice (line J9; C57BL/6 background) expressing human APP695 harboring the FAD-linked Swedish (K670N/M671L) and Indiana (V717F) mutations under the PDGF promoter were obtained by crossing APP_Sw,Ind_ to non-transgenic (WT) mice [58]. APP_Sw,Ind_-derived embryos or pups, individually genotyped and divided into “APP_Sw,Ind_” and “control”, were used for preparing primary cultures (12 embryos or pups in a typical experiment). Animal care and experimental procedures were in accordance with European and Spanish regulations (86/609/CEE; RD1201/2005). Mice were handled, as per law, by personnel with the ad hoc certificate (issued by the Generalitat de Catalunya) that allows animal handling for research purposes.

### 2.3. Neuronal and Microglial Primary Cultures

To prepare primary striatal neurons, brains from the fetuses of pregnant C57/BL6J mice were removed (gestational age: 17 days). Neurons were isolated as described in [59] and plated at a confluence of 40,000 cells/0.32 cm^2^. Briefly, the samples were dissected, carefully stripped of their meninges, and digested with 0.25% trypsin for 20 min at 37 °C. Trypsinization was stopped by adding an equal volume of culture medium (Dulbecco’s modified Eagle medium-F-12 nutrient mixture, Invitrogen). Cells were brought to a single cell suspension by repeated pipetting followed by passage through a 100 μm-pore mesh. Pelleted (7 min, 200 g) cells were resuspended in supplemented DMEM and seeded at a density of 3.5 × 10^5^ cells/mL in 6-well plates for functional assays and 12-well plates for PLA assays. For neuronal cultures, the day after, medium was replaced by neurobasal medium supplemented with 2 mM L-glutamine, 100 U/mL penicillin/streptomycin, and 2% (*v/v*) B27 (GIBCO). Primary microglia were obtained from 2–3-day-old pups and processed as described above for neurons but instead of neurobasal/B27, using Dulbecco’s modified Eagle medium supplemented with 2 mM L-glutamine, 100 U/mL penicillin/streptomycin, MEM Non-Essential Amino Acid Solution (1/100), and 10% (*v/v*) heat-inactivated Fetal Bovine Serum (FBS). All supplements were from Invitrogen (Paisley, Scotland, United Kingdom). Cells were maintained in a humid atmosphere of 5% CO_2_ at 37 °C and were used for assays after 15 days of culture. For LPS-activated microglia, 48 h before initiating the experiment, 0.01% (*v/v*) LPS and 0.002% (*v/v*) IFN-γ were added to Dulbecco’s modified Eagle medium where microglial cells were kept.

### 2.4. cAMP Determination

Microglial and neuronal primary cultures were plated in 6-well plates. Two hours before initiating the experiment, cell-culture medium was replaced by non-supplemented DMEM medium. Then, cells were detached, resuspended in non-supplemented DMEM medium containing 50 μM zardaverine, and plated in 384-well microplates (2500 cells/well). Cells were pretreated (15 min) with the corresponding antagonists (1 µM SCH 58261 for A_2A_R and 1 µM PSB 10 for A_3_R) or vehicle and stimulated with agonists (100 nM PSB 777 for A_2A_R and 100 nM 2-Cl-IB-MECA for A_3_R) (15 min) before the addition of 0.5 μM FK or vehicle. Finally, reaction was stopped by addition of the Eu-cAMP tracer and the ULight-cAMP monoclonal antibody prepared in the “cAMP detection buffer” (PerkinElmer). All steps were performed at 25°. Homogeneous time-resolved fluorescence energy transfer (HTRF) measures were performed after 60 min of incubation at RT using the Lance Ultra cAMP kit (PerkinElmer, Waltham, MA, USA). Fluorescence at 665 nm was analyzed on a PHERAstar Flagship microplate reader equipped with an HTRF optical module (BMG Lab Technologies, Offenburg, Germany).

### 2.5. MAPK Phosphorylation Assays

To determine MAP kinase 1/2 (ERK1/2) phosphorylation, primary striatal neurons and microglia were plated (50,000 cells/well) in transparent Deltalab 96-well plates and kept in the incubator for 15 days. Then, 2 h before the experiment, the medium was replaced by non-supplemented DMEM medium. Next, the cells were pre-treated at RT for 10 min with antagonists (1 µM SCH 58261 for A_2A_R and 1 µM PSB 10 for A_3_R) or vehicle and stimulated for an additional 7 min with selective agonists (100 nM PSB 777 for A_2A_R and 100 nM 2-Cl-IB-MECA for A_3_R). Then, cells were washed twice with cold PBS before the addition of 30 µL/well “Ultra lysis buffer” -PerkinElmer- (15 min treatment). Afterwards, 10 μL of each supernatant were placed in white ProxiPlate 384-well microplates and ERK1/2 phosphorylation was determined using an AlphaScreen^®^ SureFire^®^ kit (PerkinElmer) following the instructions of the supplier, and using an EnSpire^®^ Multimode Plate Reader (PerkinElmer, Waltham, MA, USA).

### 2.6. Proximity Ligation Assay (PLA)

Physical interaction between A_2A_R and A_3_R were detected using the Duolink in situ PLA detection Kit (OLink; Bioscience, Uppsala, Sweden) following the instructions of the supplier. Primary neurons and microglia were grown on glass coverslips, fixed in 4% paraformaldehyde for 15 min, washed with PBS containing 20 mM glycine to quench the aldehyde groups, and permeabilized with the same buffer containing 0.05% Triton X-100 (20 min). Then, samples were successively washed with PBS. After 1 h of incubation at 37 °C with the blocking solution in a pre-heated humidity chamber, cells were incubated overnight in the antibody diluent medium with a mixture of equal amounts of mouse anti-A_2A_R (1/100; 05-717, Millipore Corp, Billerica, MA, USA) and rabbit anti-A_3_R (1/100; ab197350, Abcam, Cambridge, United Kingdom) to detect A_2A_R-A_3_R complexes. Neurons and microglia were processed using the PLA probes detecting primary antibodies (Duolink II PLA probe plus and Duolink II PLA probe minus) diluted in the antibody diluent solution (1:5). Ligation and amplification were conducted as indicated by the supplier. Samples were mounted using the mounting medium with Hoechst (1/100; Sigma-Aldrich, St. Louis, MO, USA) to stain nuclei. Samples were observed in a Zeiss 880 confocal microscope (Carl Zeiss, Oberkochen, Germany) equipped with an apochromatic 63× oil immersion objective (N.A. 1.4) and 405 nm and 561 nm laser lines. For each field of view, a stack of two channels (one per staining) and four Z stacks with a step size of 1 μm were acquired. The number of neurons containing one or more red spots versus total cells (blue nucleus) was determined, and the unpaired t-test was used to compare the values (red dots/cell) obtained.

### 2.7. Data Handling and Statistical Analysis

Data from immunofluorescence and functional assays were analyzed using Prism 6 (GraphPad Software, Inc., San Diego, CA, USA). The data are the mean ± SEM. Statistical analysis was performed with SPSS 18.0 software. Significance was analyzed by one-way ANOVA, followed by Bonferroni’s multiple comparison post hoc test. Significant differences were considered when *p* < 0.05.

## 3. Results

### 3.1. Differential Expression of A_2A_A_3_Hets in Primary Neurons from the Cortex, Striatum, and Hippocampus

**Figure 1:**
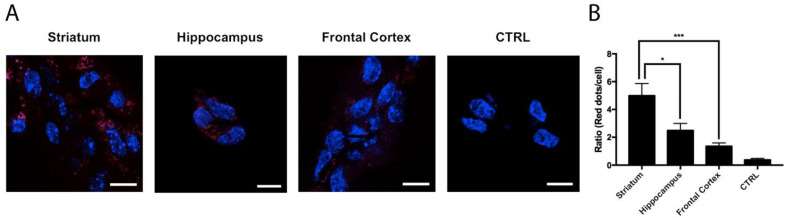
Expression of A_2A_A_3_Hets in primary cultures of neurons isolated from the fetuses of pregnant C57BL/6J female mice. (**A**) A_2A_A_3_Hets were detected by the in situ proximity ligation assay (PLA) in primary cultures of striatal, hippocampal, and cortical neurons isolated from mouse fetuses. The negative control was obtained by omitting the primary antibody anti-A_3_R. Experiments were performed in samples from 6 different animals. (**B**) The number of red dots/cell was quantified using the Andy’s algorithm Fiji’s plug-in and represented versus the number of Hoechst-stained cell nuclei (blue). The number of red dots/cell was compared to those in neurons from different brain regions. The unpaired *t*-test was used for statistical analysis. * *p* < 0.05, *** *p* < 0.001. Scale bar: 10 μm.

Using the in situ proximity ligation assay (PLA), which is instrumental to the detection of complexes formed by two proteins in natural cells and in tissue sections, the expression of A_2A_A_3_Hets was assessed in primary neurons from mice’s frontal cortex, striatum, and hippocampus. As observed in Figure 1, the expression of A_2A_A_3_Hets in the striatum was significantly higher than that in the cortex or hippocampus; these results correlate with higher expression of the heteromer in the brain region with the highest expression of A_2A_R.

### 3.2. Characterization of the Functionality of A_2A_A_3_Hets in Primary Neurons from Three Brain Regions

**Figure 2:**
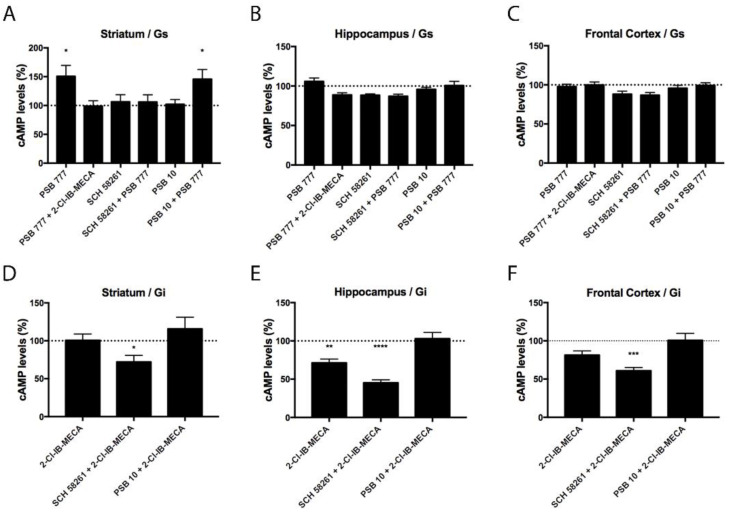
A_2A_A_3_Het-mediated Gs/Gi signaling in primary striatal, hippocampal, and cortical neurons from C57BL/6J mice. (**A**–**F**) Primary neurons were pre-treated with selective antagonists, 1 μM SCH 58261 -A_2A_R- or 1 μM PSB 10 -A_3_R-, and subsequently treated with the selective agonists, 100 nM PSB 777 -A_2A_R- or 100 nM 2-Cl-IB-MECA -A_3_R-. cAMP levels after 500 nM forskolin (FK) stimulation or vehicle treatment were detected by the Lance Ultra cAMP kit and results were expressed in % respect to basal levels (**A**–**C**) or in % respect to levels obtained upon FK stimulation (**D**–**F**). The values are the mean ± S.E.M. of 6 different experiments performed in triplicates. One-way ANOVA followed by Bonferroni’s multiple comparison post-hoc test were used for statistical analysis. * *p* < 0.05, ** *p* < 0.01, *** *p* < 0.001, **** *p* < 0.0001; versus basal levels (**A**–**C**) or versus FK treatment (**D**–**F**).

After demonstrating the differential expression of A_2A_A_3_Hets between the hippocampus, frontal cortex, and striatum, we were interested in checking whether the differential expression of the heteromer leads to differences in functionality. We have previously shown that the heteromeric context led to a marked decrease of the signaling originating at the A_3_R and that A_2A_R antagonists may overrode the blockade [9]. As the A_2A_R couples to G_s_ and the A_3_R couples to G_i_, measurement of both increases in cAMP levels and decreases of forskolin-induced cAMP levels were undertaken. In primary neurons from the striatum, where there is a higher expression of A_2A_A_3_Hets, the A_2A_R agonist, PSB 777, increased the concentration of cytosolic cAMP. The increase was only blocked by the selective A_2A_R antagonist, SCH 58261. In the striatum, the selective A_3_R agonist, 2-Cl-IB-MECA, reduced the cytosolic cAMP levels induced by PSB 777 treatment. Neither in cortical nor in hippocampal neurons did agonists lead to significant increases in cAMP levels (Figure 2A–C).

In primary striatal neurons treated with 0.5 μM forskolin (FK), the A_3_R agonist, 2-Cl-IB-MECA, was able to reduce cAMP levels only in the presence of the selective A_2A_R antagonist. This finding is consistent with previous results obtained in a heterologous expression system, namely, A_3_R activation does not lead to G_i_ engagement when the receptor is forming heteromers with A_2A_R. In contrast, the A_3_R agonist had an effect in hippocampal neurons, probably due to the expression of A_3_Rs not forming heteromers with A_2A_R. Still, the A_2A_R antagonist increased the effect of the A_3_R agonist. The results in neurons from the frontal cortex were similar, although the decrease induced by 2-Cl-IB-MECA was significant only in the presence of the A_2A_R antagonist (Figure 2D–F).

### 3.3. Characterization of the Functionality of A_2A_A_3_Hets in Primary Microglia

**Figure 3:**
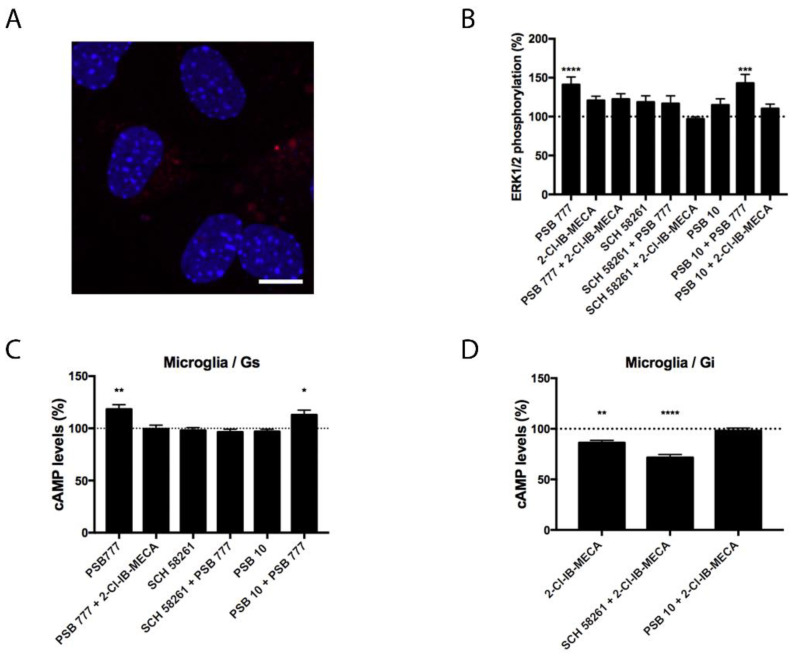
Expression and function of A_2A_A_3_Hets in primary cultures of microglia from C57BL/6J mice. (**A**) A_2A_A_3_Hets were detected in primary cultures of microglia by the in situ proximity ligation assay (PLA) using specific antibodies. Cell nuclei were stained with Hoechst (blue). Samples from 5 different animals were processed and analyzed. Scale bar: 20 μm. (**B**–**D**) Primary cultures of microglia from C57BL/6J mice were pre-treated with antagonists, 1 μM SCH 58261 -for A_2A_R- or 1 μM PSB 10 -for A_3_R-, subsequently stimulated with selective agonists, 100 nM PSB 777 -for A_2A_R- or 100 nM 2-Cl-IB-MECA -for A_3_R-, individually or in combination and treated with 500 nM forskolin (FK) or vehicle. (**B**) ERK1/2 phosphorylation was analyzed using an AlphaScreen^®^ SureFire^®^ kit (PerkinElmer) while cAMP levels were collected by the Lance Ultra cAMP kit and results were expressed in % respect to basal levels (**B**,**C**) or in % respect levels obtained upon 0.5 μM FK stimulation (**D**). Values are the mean ± S.E.M. of 10 different experiments performed in triplicates. One-way ANOVA followed by Bonferroni’s multiple comparison post-hoc tests were used for statistical analysis. * *p* < 0.05, ** *p* < 0.01, *** *p* < 0.001, **** *p* < 0.0001; versus basal (**B**,**C**) or versus FK treatment (**D**).

**Figure 4:**
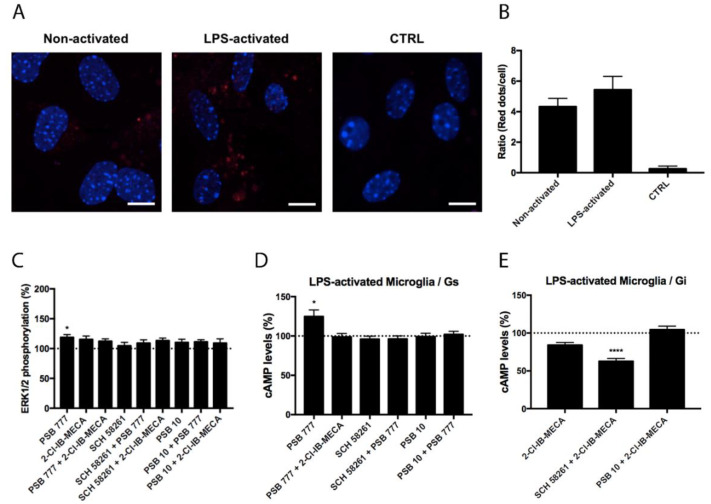
Expression and function of A_2A_A_3_Hets in primary cultures of LPS-activated microglia from C57BL/6J mice. (**A**) A_2A_A_3_Hets were detected in primary cultures of non-activated and LPS-activated microglia by the in situ proximity ligation assay (PLA) using specific antibodies. The negative control was obtained by omitting the primary anti-A_3_R antibody. Experiments were performed in samples from 5 different animals. (**B**) The number of red dots/cell was quantified using the Andy’s algorithm Fiji’s plug-in and represented versus the number of Hoechst-stained cell nuclei (blue). The number of red dots/cell was compared between non-activated and LPS-activated microglia. The unpaired t-test was used for statistical analysis. Scale bar: 20 μm. (**C**–**E**) Primary cultures of LPS-activated microglia from C57BL/6J mice were pre-treated with antagonists, 1 μM SCH 58261 -for A_2A_R- or 1 μM PSB 10 -for A_3_R-, subsequently stimulated with selective agonists, 100 nM PSB 777 -for A_2A_R- or 100 nM 2-Cl-IB-MECA -for A_3_R-, individually or in combination and treated with 500 nM forskolin (FK) or vehicle. (**C**) ERK1/2 phosphorylation was analyzed using an AlphaScreen^®^ SureFire^®^ kit (PerkinElmer) while cAMP levels were collected by the Lance Ultra cAMP kit and results were expressed in % respect to basal levels (**C**,**D**) or in % respect to levels obtained upon 0.5 μM FK stimulation (**E**). Values are the mean ± S.E.M. of 6 different experiments performed in triplicates. One-way ANOVA followed by Bonferroni’s multiple comparison post-hoc tests were used for statistical analysis. * *p* < 0.05, **** *p* < 0.0001; versus basal (**C**,**D**) or versus FK treatment (**E**).

Experiments similar to those described in the previous section were performed in primary microglia from the total brain, resting or activated using LPS and IFN-γ. Although the expression of A_2A_A_3_Hets was similar in resting versus activated cells (Figure 3 and Figure 4), the functionality of Ado receptors differed. In resting cells, the A_2A_R agonist PSB 777, was able to increase the levels of the cyclic nucleotide. This effect was completely blocked by the presence of the A_3_R agonist, showing a negative cross-talk similar to that observed in primary striatal neurons. In cells treated with FK, the effect of 2-Cl-IB-MECA was noticeable and further increased by the A_2A_R antagonist (Figure 3). Similar results were obtained in experiments performed in activated microglia; the main difference was that the significance of 2-Cl-IB-MECA on decreasing the FK-induced cAMP levels (in activated cells) required the presence of the A_2A_R antagonist (Figure 4).

Besides G_i_ or G_s_ coupling, the activation of Ado receptors leads to subsequent activation of the mitogen-activated protein kinase (MAPK) signaling pathway. To test the properties of the heteromer in the link to the MAPK signaling cascade, we measured ERK1/2 phosphorylation in resting and activated microglia. In activated cells, significant ERK1/2 phosphorylation was detected with the A_2A_R agonist; the effect did not increase when A_3_R agonist was also present (Figure 4). In resting cells, the A_2A_R agonist led to more robust ERK1/2 phosphorylation that was significantly blocked by the presence of the A_3_R agonist without any significant change in the presence of the A_3_R antagonist (Figure 3). Interestingly, the A_3_R agonist induced no effect in MAPK phosphorylation. Contrary to the data observed in the cAMP assays, the pretreatment with A_2A_R antagonists never potentiated this effect.

### 3.4. Expression and Functionality of the Heteromer in the APP_Sw,Ind_ Transgenic Mice Model of Alzheimer’s Disease (AD)

**Figure 5:**
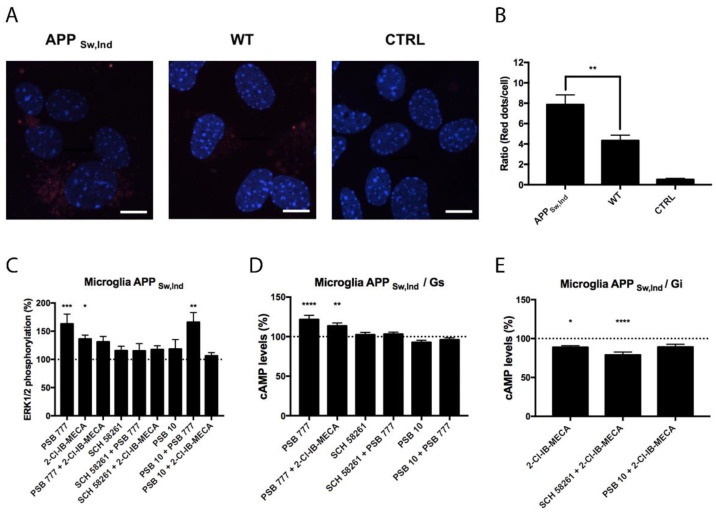
Expression and function of A_2A_A_3_Hets in primary cultures of microglia from the APP_Sw,Ind_ mice model of Alzheimer’s disease. (**A**) A_2A_A_3_Hets were detected in primary cultures of APP_Sw,Ind_ microglia by the in situ proximity ligation assay (PLA) using specific antibodies. The negative control was obtained by omitting the primary anti-A_3_R antibody. Experiments were performed in samples from 12 different animals. (**B**) The number of red dots/cell was quantified using the Andy’s algorithm Fiji’s plug-in and represented versus the number of Hoechst-stained cell nuclei (blue). The number of red dots/cell was compared to those in microglia from wild type (WT) mice. The unpaired t-test was used for statistical analysis. ** *p* < 0.01, versus WT. Scale bar: 20 μm. (**C**–**E**) Primary cultures of microglia from APP_Sw,Ind_ mice were pre-treated with antagonists, 1 μM SCH 58261 -for A_2A_R- or 1 μM PSB 10 -for A_3_R-, and subsequently stimulated with selective agonists, 100 nM PSB 777 -for A_2A_R- or 100 nM 2-Cl-IB-MECA -for A_3_R-, individually or in combination. (**C**) ERK1/2 phosphorylation was analyzed using an AlphaScreen^®^ SureFire^®^ kit (PerkinElmer) while cAMP levels were collected by the Lance Ultra cAMP kit and results were expressed in % respect to basal levels (**C**,**D**) or in % respect levels obtained upon 0.5 μM FK stimulation (**E**). Values are the mean ± S.E.M. of 6 different experiments performed in triplicates. One-way ANOVA followed by Bonferroni’s multiple comparison post-hoc tests were used for statistical analysis. * *p* < 0.05, ** *p* < 0.01, *** *p* < 0.001, **** *p* < 0.0001; versus basal (**C**,**D**) or versus FK treatment (**E**).

Microglial cells are important in neurodegenerative diseases coursing with neuroinflammation. For this reason, we compared the expression of A_2A_A_3_Hets in primary microglia from the brain of the APP_Sw,Ind_ transgenic AD model and of age-matched control mice. Figure 5A,B shows that the expression of the heteromer was increased by about two-fold in cells from the transgenic animal. Functionality was assessed in cells from the APP_Sw,Ind_ mice by measuring cAMP and pERK1/2 levels upon stimulation with agonists. The G_s_ coupling was confirmed when the A_2A_R agonist, PSB 777, was used. This effect was counteracted when cells were coactivated with the A_3_R agonist and PSB 777, showing a negative cross-talk, and also when preactivated with the A_3_R antagonist, showing cross-antagonism (Figure 5D), which was also found in activated cells from control mice (Figure 4D). Regarding G_i_ coupling, we found a significant effect of the A_3_R agonist, 2-Cl-IB-MECA, that was further enhanced by the A_2A_R antagonist, SCH 58261 (Figure 5E). The level of ERK1/2 phosphorylation was much higher when cells were treated with the A_2A_R agonist than when cells were treated with the A_3_R agonist. Costimulation led to a negative cross-talk, i.e., to a reduced signal when compared to the effect exerted by the A_2A_R agonist. Importantly, PSB 10 did not reduce the effect of the A_2A_R agonist, i.e., no cross-antagonism from A_3_R to A_2A_R was detected in these cells.

## 4. Discussion

The results in this paper confirm a previous report showing that A_2A_R and A_3_R interact to form heteromeric complexes [9]. These complexes occur naturally and their expression in primary neurons correlates with the expression of A_2A_R in different brain regions. In fact, the expression of A_2A_A_3_Hets was much higher in neurons from the striatum than from the cortex or hippocampus (Figure 1).

There is interest in the potential of targeting A_2A_Rs for combating a variety of diseases, especially after the success in launching istradefylline, a selective A_2A_R antagonist for adjuvant therapy of Parkinson’s disease. A_2A_R antagonists are also promising candidates to combat AD. In fact, work in transgenic animal models has shown neuroprotective activity and/or reversal of cognitive impairment [22,25,60,61,62,63,64]. Research on AD has focused mainly on neurons and this has revealed that extracellular deposits of amyloid and intraneuronal deposits of aberrantly phosphorylated tau protein are the two main pathological hallmarks of the disease. Antagonists of A_2A_R may afford neuroprotection by preventing the noxious actions of ß-amyloid peptide, as shown in cultured neurons and isolated nerve terminals [62,65]. Although A_3_R in the CNS has received less attention than other types of ARs, Stone suggested in 2002 that chronic administration of A_3_R agonists may provide neurons with protection against damage [66]. In summary, blocking A_2A_R or activating A_3_R in neurons seem to be neuroprotective in pathological scenarios. The discovery of A_2A_A_3_Hets and of the negative functional cross-talk [9] enables neuroprotection by A_2A_R antagonists targeting neurons expressing these heteromers. Indeed, within the heteromer, A_2A_R antagonists would block A_2A_R while making A_3_R respond to endogenous adenosine, i.e., exogenous A_3_R agonists would not be necessary to provide neuroprotection.

Neuroprotection—that is, addressing disease progression—may likely be achieved by targeting the glia that surround neurons whose survival is already significantly compromised. Microglia are attracting attention in the field of AD because microglial cells are involved in the inflammation that occurs in the brain of patients and because microglia could acquire a neuroprotective phenotype, generally known as M2 as opposed to M1 or pro-inflammatory; among the several GPCRs expressed in these cells, A_2A_R is an attractive target to regulate microglial activation and polarization [30,33,40,67,68,69,70,71,72,73,74,75,76,77].

A_2A_R seems to have a residual role in resting microglia, but it becomes a main player in AD-related activated microglia, pointing to the A_2A_R expressed in these cells as an attractive target. Adenosine regulates microglial fate and activation [42,47,78,79,80], by unknown mechanisms and without details on the role of each AR type on cell polarization. The microglial A_2A_R regulates lipopolysaccharide-induced neuroinflammation [45] and further data on the potential of these glial cells in the context of neurodegeneration and neuronal dysfunction was provided in 2016 by Cunha [75]. Despite the few reports on A_3_R and microglia, the receptor is expressed [44,81] in primary cultures and immortalized microglial cells; apart from its contribution to the MAPK signaling pathway [44], it mediates the inhibition of cell migration [54,82,83] and the production of TNFα [50].

In previous reports, we have shown that A_2A_R regulates nitric oxide production in cells activated with LPS and IFN-γ [39]. Moreover, A_2A_R is markedly upregulated in the cortical and hippocampal microglia of AD patients [26]. Therefore, it is relevant to consider how the interaction of A_2A_R with other cell surface proteins can affect its functionality in both healthy and pathological conditions. Previous reports have demonstrated in microglia that A_2A_R interacts with the cannabinoid CB_2_ receptor to form functional units in which cannabinoid signaling is compromised; however, the presence of A_2A_R antagonists leads to potentiation of cannabinoid signaling. These functional complexes are up-regulated in the APP_Sw,Ind_ AD transgenic mice model [33], thus reinforcing the view that A_2A_R antagonists may be useful in the therapy of AD. Additionally favoring the potential of A_2A_R antagonist in AD therapy is the reduced activity of N-methyl-D-aspartate receptors (NMDARs), resulting from cross-antagonisms within macromolecular complexes formed by NMDA and A_2A_ receptors in both neurons and microglia [32]. It should be noted that one of the current anti-AD treatments consists of reducing NMDAR function by using an allosteric negative modulator (memantine) [84].

Functional data in neurons and microglia are partly similar to those found in a heterologous system in which the two receptors are expressed and form A_2A_A_3_Hets. Hence, A_3_R-mediated signaling seems to be reduced/absent except in the presence of A_2A_R antagonists. Regarding A_2A_R-mediated signaling, we found that the use of the more selective agonist, PSB 777, leads to a reduced signal if compared to that reported using CGS 21680. At first sight, this is puzzling as PSB 777 is a very selective and full agonist of A_2A_R [85]; however, differential selectivity depending on the context may be the answer. The more robust signaling using CGS 21680 is not likely due to binding to A_3_R as the use of the selective A_3_R agonist, 2-Cl-IB-MECA, does not lead to significant responses either. This fact also suggests that PSB 777, in the experimental conditions and models used here, is not specially biased. In conclusion, it seems that PSB 777 is less potent than CGS 21680 in activating G_s_-mediated signaling or in the link to the MAPK pathway cascade in microglia [9,33]. Functional selectivity in the form of biased agonism or else is dependent on the context of GPCR, especially if the receptor is part of a heteromer. Our results confirm that signaling via A_2A_R or A_3_R varies depending on the context, that is, on the structure of the heteromer and/or allosteric interactions with G and scaffold proteins [86,87]. Otherwise, it would be difficult to interpret why the signal is more robust in resting than in activated microglia although the expression of the heteromer is similar. Even assuming that part of the signaling is independent of A_2A_A_3_Het, the effect of PSB 777 should, theoretically, be higher in activated microglia where A_2A_R is upregulated.

In conclusion, it is important to note that A_2A_A_3_Het is upregulated in the APP_Sw,Ind_ AD model and that signaling through A_2A_R may depend on its interaction with other cell surface proteins and/or with proteins of the signaling machinery. In the primary microglia of the APP_Sw,Ind_ AD model, the functional selectivity is such that A_2A_R would increase G_i_-mediated signaling of A_3_R. These findings reinforce the idea that A_2A_R antagonists deserve consideration to combat AD.

## Data Availability

Data are available upon reasonable request to corresponding authors.
